# Distinct histological patterns in chronic hepatitis D with nucleos(t)ide analogue therapy

**DOI:** 10.3389/fmed.2023.1082069

**Published:** 2023-04-06

**Authors:** Julian Hercun, Theo Heller, Jeffrey S. Glenn, David E. Kleiner, Christopher Koh

**Affiliations:** ^1^Translational Hepatology Section, Liver Diseases Branch, National Institute of Diabetes and Digestive and Kidney Diseases, National Institutes of Health, Bethesda, MD, United States; ^2^Departments of Medicine (Division of Gastroenterology and Hepatology) and Microbiology & Immunology, Stanford University School of Medicine, Stanford, CA, United States; ^3^NCI Laboratory of Pathology, National Institutes of Health, Bethesda, MD, United States

**Keywords:** viral hepatitis, hepatitis D (delta) virus, nucleoside analog, histology, hepatitis B virus

## Abstract

**Background:**

Chronic hepatitis delta virus (HDV) infection leads to a more severe hepatitis than hepatitis B virus (HBV) infection alone. Specific histological staining patterns have been described in HBV mono-infection, however this has not been extensively investigated in HDV co-infection. This study evaluated whether the use of nucleos(t)ide analogs (NAs) for concurrent HBV infection has an impact on the histological appearance of chronic HDV.

**Methods:**

Liver biopsies of all patients referred for management of HDV infection were reviewed and hepatitis-specific stains for HBV antigens were evaluated. Clinical and histological characteristics were compared between patients on and off-NA therapy.

**Results:**

50 patients were included in our analysis, of which 26 (52%) were on NA therapy at the time of the biopsy. Overall, 8% stained for HBV core antigen and 86% stained for HBV surface antigen. On and off-NA groups had similar degrees of fibrosis and inflammation, however NA patients had an odds ratio of 7.15 for membranous staining and 0.13 for scattered granular staining (*p* = 0.001). No association was found with markers of disease severity or viral activity, with nonetheless a lower score of total inflammation noted in biopsies with a positive membranous stain (8.5 vs. 10.3 *p* = 0.04).

**Conclusion:**

In chronic HDV infection, patients treated with nucleos(t)ide analogs demonstrate a unique membranous staining pattern for hepatitis B surface antigen, which is not associated with HBV or HDV replicative activity. These findings may help improve the understanding of the role of HBV directed therapy in HDV pathophysiology.

**Highlights:**

- Histological staining is associated with viral activity in chronic HBV, however this has been infrequently explored in HDV. In HDV, staining patterns differ based on HBV treatment status and do not appear to be associated with markers of viral activity.

## Introduction

Chronic hepatitis delta virus (HDV) infection occurs only in patients with underlying hepatitis B infection (HBV) and leads to a more severe form of hepatitis, a more rapid progression to cirrhosis and an increased risk of hepatocellular carcinoma compared to HBV infection alone (1). Interferon-alfa based therapies, while not approved by the US Food and Drug Administration, are currently the only widely available treatments for HDV endorsed by major societies, but are plagued by low tolerability and sub-optimal results. The judicious use of nucleos(t)ide analogs (NA), pillars in the current treatment of HBV, has been integrated in practice guidance for HDV (2, 3). However NAs have been shown to be ineffective in controlling HDV viremia when used as mono-therapy and do not improve response to interferon (4, 5). Furthermore, the use of NAs in chronic HDV does not reduce the occurrence of hepatic decompensation, hepatocellular carcinoma, liver transplantation or death (6, 7).

Histologically, HDV is associated with a more severe liver injury and more active hepatitis than HBV infection alone (8). Specific histological staining patterns have not been extensively investigated in chronic HDV co-infection. In HBV mono-infection, HBV surface antigen (HBsAg) staining patterns correlate with the stage of disease and activity (9). Both nuclear HBV core antigen (HBcAg) expression and membranous HBsAg have been associated with viral replication (10–12).

In HDV, patterns of expression of hepatic HBV antigens are affected by viral interference and suppression of HBV replication. Overall, in co-infected patients, a smaller proportion of cases stain positive for HBV antigen stains (13, 14). Magnitude of HBsAg staining has also been associated with stages of liver disease in HDV (15). Hepatic expression of hepatitis delta antigen has been extensively described, correlating with the extent of inflammation on biopsy (16). However, while it is known that HDV and HBV antigens coexist in the liver (17), very few studies have evaluated the impact of HBV treatment on patterns of hepatic expression of HBV antigens in delta hepatitis. This study evaluated whether the use of NAs for concurrent HBV infection had an impact on the histological appearance of chronic HDV.

## Materials and methods

Chronic HDV patients having undergone liver biopsy for evaluation and staging of liver disease at the National Institutes of Health between 2000 and 2020 were considered for analysis. Chronic HDV infection was determined by confirmation of the presence of HBsAg as well as anti-HDV antibodies in the serum and a positive delta antigen stain on liver histology and/or a quantifiable serum HDV RNA for more than 6 months.

Patients on treatment with Nucleos(t)ide analogs (Entecavir or Tenofovir) within the 6 months prior to the biopsy were considered as HBV on-treatment patients. All clinical characteristics were taken at the time of the biopsy, and laboratory test within 6 months from the biopsy were considered for analysis. All the data was collected from timepoints before the introduction of any additional HDV treatment with the exception of pegylated interferon. Recent interferon use was defined as within 6 months prior to biopsy. Patients treated in the context of clinical protocols were only included if biopsy was performed 6 months or more after withdrawal of HDV-directed therapy.

All biopsies were reviewed by an expert hepatopathologist (DEK) and biopsies performed both at the NIH Clinical Center and at outside centers were evaluated. Fibrosis stage was scored using Ishak fibrosis score and inflammation was assessed using the modified Histology Activity Index (HAI) on hematoxylin and eosin stain (18). All biopsies were processed in a similar fashion and hepatitis-specific stains for Hepatitis B surface antigen (HBsAg), Hepatitis B core Antigen (HBcAg) and Hepatitis D antigen (HDAg) were obtained. HBsAg (Thermo predilute, clone 3E7) and HBcAg (Dako B0586, 1:500) stains were performed on a Ventana Benchmark Ultra. HDAg stains were performed by manual staining using a high titer patient serum diluted at 1:1000 and detected using a biotinylated goat antihuman IgG (1:200, Vector Laboratories). Detection was performed using an avidin–biotin complex and diaminobenzidine chromagen (19). Four patterns of HBsAg staining were scored: inclusion-like, scattered granular, contiguous granular and membranous (all scored present or absent) (20). The HBsAg, HBcAg and HDAg stains were graded semi-quantitively on a scale of 0 to 3 (respectively 0%, <10%, 10–50 and > 50%) based on the proportion of the parenchymal staining. The overall HBsAg staining percentage was also noted, excluding the percentage of membranous staining which was reported separately. In addition to semi-quantitative grade for core-antigen, the proportion of nuclear and cytoplasmic proportion was estimated. Cases where HBsAg and HBcAg staining was inadequate were not considered in this analysis. Only initial biopsies were included in the overall analysis and follow-up liver biopsies (whenever available) were considered in a sub-analysis.

HDV RNA was measured using quantitative PCR with an assay from ARUP Laboratories, (Salt Lake City, UT, United States) (lower limit of detection 120 IU/mL), HBV DNA was detected using Roche COBAS® AmpliPrep/COBAS® TaqMan® HBV Test, version 2.0 (lower limit of detection of 20 IU/mL) and quantitative HBsAg levels were measured with an assay from The Doctors Laboratory (TDL) (London, United Kingdom) (lower limit of detection 0.05 IU/mL).

Association between all clinical, laboratory and histological variables and staining patterns between the on and off-treatment groups was investigated. Correlation was evaluated using Spearman’s rank correlation. Continuous variables were evaluated through *t*-tests or equivalent non-parametric tests (Wilcoxon rank sum test). Categorical variables were evaluated by Chi-squared or Fisher’s exact test. Logistic regression was used to assess strength of association. *p* values <0.05 were considered significant. All data analysis was performed using R (Version 3.6.1).

Preparation and reporting of this manuscript adhered to the STROBE guidelines. All patients were enrolled in clinical research protocols conforming to the 1975 Declaration of Helsinki and approved by the National Institute of Diabetes and Digestive and Kidney Diseases Institutional Review Board and gave written informed consent for participation.

## Results

### Patient population

**Figure 1:**
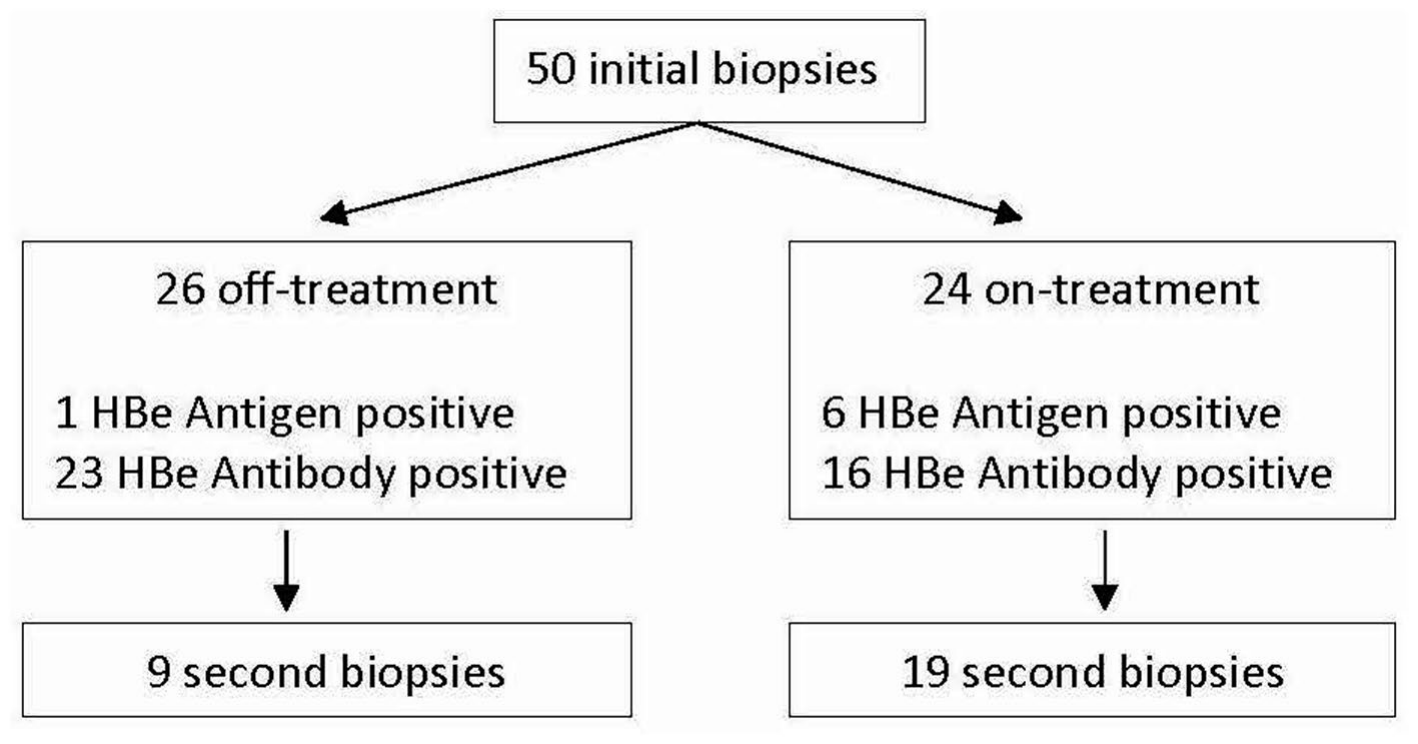
Study flow diagram.

50 patients, 68% male with a mean age of 40.7 years at the time of the biopsy were included in our analysis. 24 (48%) were on NA therapy at the time of the biopsy. While 21 patients had previously received additional HDV-directed treatment, only 7 patients received Interferon in the 6 months prior to the biopsy. 7 patients (14%) were HBe antigen (HBeAg) positive, while 39 (78%) were Anti-HBe Antibody positive (Figure 1) Mean HBV DNA was 19,600 international units (IU)/mL (Standard deviation (SD) 130,000), undetectable in 25 patients (50%), however with only two patients with an HBV DNA > 2000 IU/mL. Mean HDV RNA was 4.8 log_10_ IU/mL(SD 1.9) and was higher in the HBeAg positive subgroup (6.2 compared to 4.6 log_10_ IU/mL, *p* = 0.012). No differences in baseline serum HBsAb titers and HBV DNA were noted based on HBeAg status. Baseline mean ALT was 120 U/L, AST 84 U/L, total bilirubin 0.7 mg/dL and platelet count 158 × 10^9^/L. Additional baseline characteristics are described in Table 1.

**Table 1:** Baseline characteristics of the overall cohort and stratified by treatment status.

	Overall cohort	Off treatment	On treatment	*p* value
	(*n* = 50)	(*n* = 26)	(*n* = 24)	
Age (years)	40.7 (11.3)	41.7 (11.9)	39.56 (10.8)	0.50^c^
Race % (*n*)	Asian 52% (26)	Asian 42% (11)	Asian 63% (15)	0.29^a^
	White 40% (20)	White 50% (13)	White 29% (7)	
	Black/African-American 8% (4)	Black/African-American 8% (2)	Black/African-American 8% (2)	
Gender (Male) - %(*n*)	68% (34)	69% (18)	67% (16)	0.84^b^
Previous treatment - %(*n*)	42% (21)	42% (11)	42% (10)	0.96^b^
Recent Interferon treatment (<6 months) - %(*n*)	14% (7)	23% (6)	4% (1)	0.10^a^
Transient Elastography (kPa)	11.3 (7.0)	11.8 (8.0)	10.9 (6.5)	0.78^d^
Platelet count (x 10^9^/L)	159 (61.7)	146 (66.8)	173 (53.7)	0.13^c^
GGT (U/L)	73 (73.3)	**83 (55.9)**	**63 (89.3)**	**0.03** ^d^
ALP (U/L)	85(40.4)	86 (35.1)	85 (46.3)	0.73^d^
AST (IU/L)	84 (67.6)	82 (52.3)	86 (82.3)	0.49^d^
ALT (IU/L)	120 (129.0)	114 (97.7)	127 (158.3)	0.98^d^
Albumin (g/dL)	3.9 (0.5)	3.8 (0.4)	4.1 (0.5)	0.08^c^
Total bilirubin (mg/dL)	0.7 (0.5)	0.7 (0.2)	0.8 (0.7)	0.68^d^
PT (sec)	14.0 (1.0)	14.0 (1.2)	13.9 (0.8)	0.65^d^
HBV DNA (IU/mL)	19,600 (130400)	38,280 (182400)	57 (117)	0.26^d^
Undetectable serum HBV DNA-% (*n*)	50% (25)	48% (12)	52% (13)	0.57^b^
HDV RNA (IU/mL)	1,337,200 (2715200)	1,370,420 (3362480)	1,301,000 (1847160)	0.10^d^
Log HDV RNA (log_10_ IU/mL)	4.80 (1.90)	4.20 (2.30)	5.50 (1.00)	0.10^d^
Quantitative HBs Ag (IU/mL)	14,690 (14450)	13,750 (14720)	15,590 (14460)	0.59^d^
HbeAg positive %(*n*)	14% (7)	**4% (1)**	**25% (6)**	**0.045** ^a^
HbeAb positive % (*n*)	78% (39)	88% (23)	67% (16)	0.06^b^
Ishak fibrosis	3.4 (1.7)	3.9 (1.6)	2.9 (1.7)	0.054^d^
HAI Score	9.6 (2.6)	9.8 (3.0)	9.5 (2.2)	0.63^c^
Hepatitis D Antigen stain	1.3 (0.6)	1.2 (0.4)	1.4 (0.7)	0.24^d^

Continuous variables are expressed as Mean (Standard deviation).

a Fisher’s exact test.

b Chi squared.

c Independent *t*-test.

d Wilcoxon rank sum test. Bold = *p* value < 0.05.

**Figure 2:**
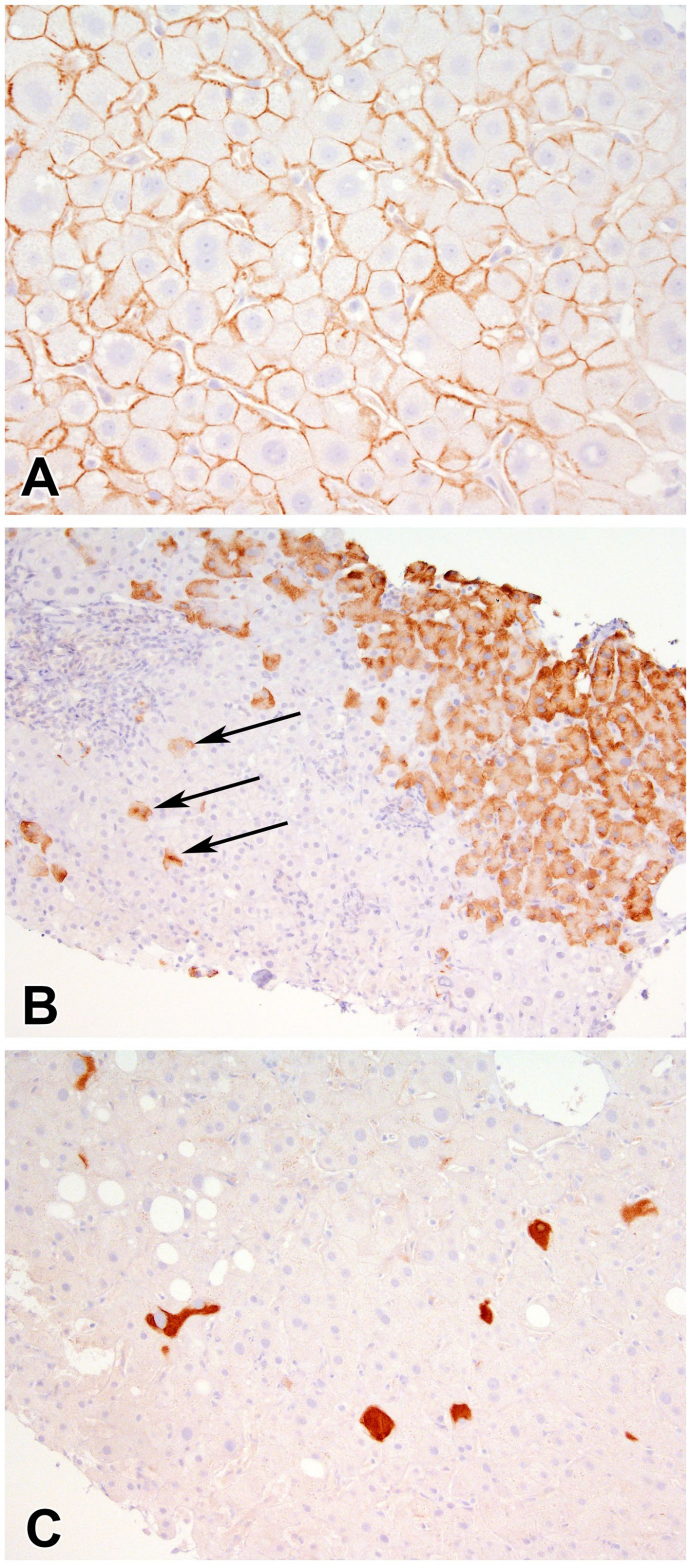
Immunohistochemical stain for Hepatitis B Surface antigen showing **(A)** membranous staining (400x), **(B)** scattered granular (arrows) and contiguous granular cytoplasmic staining (400X), and **(C)** inclusion-like (200X) staining of hepatocytes.

In the overall study cohort, mean Ishak fibrosis score was 3.4 (SD 1.7), 14 patients (28%) had cirrhosis and mean total inflammation HAI score was 9.6 (SD 2.6). Overall, 8% of biopsies had a positive HBcAg stain and 86% expressed HBsAg. On average 30% of hepatocytes stained positively for HBsAg. In HBsAg positive samples, inclusion-like, scattered granular, contiguous granular and membranous patterns (Figure 2) were present in 77, 74, 72 and 40% of cases, respectively. In patients expressing a membranous pattern, an average of 75% of the hepatocytes stained in this pattern. In the 43 biopsies with available HDAg staining, 98% were positive.

### Surface antigen staining associations with disease activity

There was no correlation between Hepatitis B surface antigen staining (in percentage) and markers of serum viral activity including serum HBsAg titers (*r* = 0.29, *p* = 0.09), HDV RNA (*r* = 0.29, *p* = 0.07), HBV DNA (*r* = −0.17, *p* = 0.27). Similarly, there was no correlation with serological markers of inflammation ALT (*r* = −0.28, *p* = 0.08), AST (*r* = −0.23, *p* = 0.15) or histological inflammation and HAI (*r* = −0.22, p = 0.15). Out of the four biopsies expressing HBcAg, overall staining was weak with nuclear expression in 0.25% and a cytoplasmic expression in 7.5% of hepatocytes.

### Comparison between on and off-treatment groups

Baseline characteristics were comparable between both on and off-treatment groups (24 on-treatment and 26 off-treatment) and are presented in Table 1. There was no significant difference in previous treatment (either HBV treatment or interferon) as well as recent Interferon use. Liver enzymes were comparable between both groups although GGT levels were higher in the on-treatment group (83 vs. 63 U/L) (*p* = 0.04). Additionally, there was a higher prevalence of HBeAg positivity in the on-treatment group (25 vs. 3%) (*p* = 0.045). HBV and HDV viral loads, as well as histological scores of fibrosis and inflammation were not statistically different between groups.

Both on and off-NA groups had similar overall fraction of hepatocytes positive for HBsAg and HBcAg. However, patterns of HBsAg staining were different between on and off-NA patients and are presented in Table 2. A greater number of off-NA cases expressed a scattered granular pattern (59 vs. 91% *p* = 0.014) while a greater number of on-NA cases expressed a membranous pattern (59 vs. 19% *p* = 0.007). No difference was noted in the proportion of inclusion-like and contiguous staining patterns between the groups. With both staining patterns considered together, on-NA patients had an odds ratio of 7.15 for membranous staining and 0.13 for scattered granular staining (model *χ*^2^ (2) =13.52, *p* = 0.001). This remained significant when controlled for recent Interferon use and HBeAg positivity, with an odds ratio of 12.03 for membranous staining and 0.03 for scattered granular staining in NA patients (model *χ*^2^(4) = 28.26, *p* < 0.001).

**Table 2:** Hepatitis B histological staining patterns compared between On and Off treatment groups.

	Overall cohort	Off treatment	On treatment	*p* value
	(*n* = 50)	(*n* = 26)	(*n* = 24)	
Surface Antigen positive % (*n*)	86% (43)	81% (21)	92% (22)	0.19^c^
Surface Antigen score (mean-SD)	1.6 (1.0)	1.4 (0.9)	1.8 (1.1)	0.11^b^
Score 0	7	5	2	
Score 1	20	9	11	
Score 2	11	10	1	
Score 3	12	2	10	
Surface Antigen % hepatocytes (mean-SD)	30% (32)	20% (18)	40% (38)	0.21^b^
Inclusion positive % (*n*)	77% (33)	67% (14)	86% (19)	0.16^c^
**Scattered granular positive % (*n*)**	74% (32)	**91% (19)**	**59% (13)**	**0.02** ^a^
Contiguous granular positive % (n)	72% (31)	62% (13)	82% (18)	0.15^a^
**Membranous positive % (*n*)**	40% (17)	**19% (4)**	**59% (13)**	**0.007** ^a^
Membranous % hepatocytes (mean-SD)	75% (29)	63% (3)	79% (26)	0.35^b^
Core Antigen score (mean-SD)	0.1 (0.4)	0.2 (0.5)	0.04 (0.2)	0.36^b^

SD: Standard deviation.

a Chi squared.

b Wilcoxon rank sum.

c Fishers exact test.

Bold = *p* value < 0.05.

### Associations with membranous staining pattern

No correlation was found between membranous staining intensity (by percentage) and the following markers of viral activity: HDV RNA (*r* = 0.11, *p* = 0.68), HBV DNA (*r* = 0.31, *p* = 0.23) and HBsAg levels (*r* = 0.09, *p* = 0.73). In addition, no correlation was noted with histological inflammation and fibrosis scores or the following biochemical markers: ALT, AST, ALP, GGT and platelets. When comparing cases with positive or negative membranous staining, there was no significant difference in serum levels of HDV (*p* = 0.70), HBV (*p* = 0.96), HBsAg (*p* = 0.11) and biochemical markers ALT (*p* = 0.19), AST (*p* = 0.29), ALP (*p* = 0.63), GGT (*p* = 0.15), total bilirubin (*p* = 0.99), and platelet counts (p = 0.63). However, while there was no difference in histological fibrosis (*p* = 0.25), a positive membranous stain was associated with a significantly lower HAI score (8.5 vs. 10.3 *p* = 0.04).

### Associations with follow-up biopsy

In 28 patients, a follow-up liver biopsy performed on average 29 months after the initial biopsy (SD 32 months) was available. 68% of patients with a follow-up biopsy were treated with NAs at the time of the original biopsy. Overall, 89% stained positive for HBsAg and none for HBcAg. HBsAg staining remained stable over time in individual biopsies from first to second biopsy (*p* = 0.22) with an average of 24% of hepatocytes staining positive on follow-up biopsy.

Surface antigen staining patterns remained consistent over time, with the proportion of biopsies staining positive each pattern remaining identical; 88% had an inclusion-like pattern, 92% a scattered granular pattern, 72% a contiguous granular pattern and 68% a membranous pattern. Fisher’s exact test non-significant for all four staining patterns. 8 patients (29%) expressed an identical staining pattern on repeat biopsy. Overall, magnitude of membranous staining remained stable over time in the entire cohort (79% vs. 69%, *p* = 0.7) and did not differ in individual biopsies (*p* = 0.52). This stability in membranous staining over time was also seen in the sub-group already on NAs at the time of the first biopsy (*p* = 0.81). In the sub-group of patients in which NA therapy was introduced in between both biopsies (*n* = 7), 60% presented a scattered granular pattern with negative membranous staining, which was not seen in the follow-up biopsies. Furthermore, membranous staining was expressed in 67% of cases.

## Discussion

In this retrospective analysis of 50 patients with chronic HDV infection, a unique HBsAg staining pattern differentiating patients on and off-treatment with nucleos(t)ide analogs for HBV is described. Overall, a minority of cases (8%) stain positive for HBcAg, however a majority of cases (86%) stain positive for HBsAg. While 40% of the study cohort expresses membranous HBsAg staining, a significant difference between patients on and off-NAs was observed with an odds ratio of 7.15 for membranous staining in treated patients. In addition, we demonstrate that HBsAg staining patterns remain stable on subsequent biopsies.

While it was initially thought that patients with delta hepatitis would exclusively stain for the delta antigen, additional studies described coexistence of both HBV antigens in chronic HDV patients (13, 17). Overall, in cohorts of HBV mono-infected patients, HBcAg is detected in 47–55% of hepatocytes, and HBsAg in 97% (with membranous expression in 41.5%) (21, 22). In HDV co-infection, intensity of HBsAg staining has been linked to earlier stages of disease and HBcAg positivity rates are low (15). The results from our cohort are consistent with previous reports of untreated chronic HDV patients, in which 6% of patients stained positive for HBcAg and 79.6% for HBsAg (14).

In our cohort, the low percentage of HBcAg staining is consistent with the low levels of HBV viral expression. Absence of HBcAg in the liver has previously been associated with low levels of HBV viral replication and minimal inflammatory activity (14, 23). A higher prevalence of HBcAg has been noted in the presence of higher viral loads in these cohorts (24). In the chronic HDV population, suppression of HBV replication is due to active HDV infection in addition to potential use of HBV therapy. In our cohort, the treated population includes non-cirrhotic patients who are either immune active patients or pre-core mutants; therefore a higher number of HBeAg positive cases in the on-NA group is expected. This also explains the lack of association with HBV viral activity markers in our subgroup of HBeAg positive patients.

In HBV, membranous HBsAg expression has been associated with active viral replication, and immune-mediated response (21, 25). However, other studies have not found a correlation between histological inflammatory activity and HBsAg staining intensity or pattern in HBV (26). In HDV co-infection, a previous study reported that membranous expression was seen less frequently in HDV positive patients than in HBV mono-infected patients (58% vs. 94% of cases) (27). The results from our study demonstrate a correlation between NA treatment and membranous staining without correlation to markers of HBV or HDV viral activity, a finding in line with previous descriptions of patterns of hepatitis delta antigen staining (16).

In addition to the findings regarding membranous staining, the overall prevalence of a scattered granular pattern in this cohort is reflective of persistent HBsAg positivity in all patients. In our cohort, no difference in serum quantitative hepatitis B surface antigen was noted based on NA status. Surface antigen levels remained elevated even in the context of suppressed HBV DNA. In a previous study, once stratified for histological severity of liver disease, no difference with serum titers of HBsAg was found between HBV mono-infected and HDV patients (14).

Patterns of staining in HDV patients in response to treatment have not been studied extensively. After interferon treatment in HBV, the HBsAg staining pattern has been shown to evolve from a cytoplasmic/inclusion pattern to a predominantly marginal/negative pattern (28). In a HBV/HDV co-infected cohort, a decrease in intensity of HBsAg and HBcAg was noted at the conclusion of interferon therapy (15). No differences in HBsAg and HBcAg expression in the liver were noted in a subgroup of patients after adefovir treatment (15). Our study adds to the body of evidence regarding hepatic expression of HBV antigens in chronic HDV infection and describes a pattern of staining associated with NA use. Furthermore, this association remains significant even when recent interferon use is taken into account. This finding could be reflective of the diversion of the HBV surface antigen toward HDV assembly in cases where the HBV polymerase is inhibited. This particular staining pattern can represent aborted viral replication, residual production from integrated HBV DNA and persistence of covalently closed circular DNA, stages in the infectious cycle which are not targeted by NAs (29).

This study is not without limitations. Due to the relative rarity of chronic HDV in most clinical settings as well as the heterogeneous presentation of liver disease in this population, a controlled comparison with HBV mono-infected patients and stratified on severity of liver disease, viral activity and treatment status was not feasible. In addition, histopathological assessment of viral hepatitis can potentially be influenced by sampling error and the sensitivity of assays. In addition, due to the retrospective nature of this cohort, assessment of serum and intrahepatic viral activity through novel assays could not be performed. Furthermore, impact of duration of NA therapy could not be assessed, as therapy was often initiated before referral to our center. Nonetheless, our sizeable real-world cohort benefits from an extensive virological assessment and thorough histological analysis. Our results highlight that the histopathological evaluation of HDV cannot be interpreted solely like HBV and that treatment can modulate histological expression.

In conclusion, use of HBV nucleo(s)tide analog therapy in chronic HDV infected patients resulted in a unique Hepatitis B surface antigen membranous staining pattern. No association between serological markers of disease activity and membranous staining pattern was found in this HDV cohort, which differs from previous reports in HBV mono-infection. The use of HBV suppressive therapy may have allowed for indirect evidence of diversion of HBV surface antigen toward HDV replication, a finding not previously seen histologically. Further understanding of the role of nucleos(t)ide analog therapy in HDV may allow for an improved understanding of the pathophysiology of HDV infection.

## Data availability statement

The raw data supporting the conclusions of this article will be made available by the authors, without undue reservation upon written request.

## Ethics statement

The studies involving human participants were reviewed and approved by National Institutes of Health Institutional Review Board (IRB). The patients/participants provided their written informed consent to participate in this study

## Author contributions

JH, DEK, and CK: study conception and design. JH, JG, DEK, and CK: acquisition of data. JH, TH, JG, and DEK: analysis and interpretation of data. JH, DEK, and CK: drafting of the manuscript. JH, TH, JG, DEK, and CK: critical revision of the manuscript for important intellectual content. All authors contributed to the article and approved the submitted version.

## Funding

This work was supported by the NIDDK Intramural Research Program.

## Conflict of interest

The authors declare that the research was conducted in the absence of any commercial or financial relationships that could be construed as a potential conflict of interest.

## Publisher’s note

All claims expressed in this article are solely those of the authors and do not necessarily represent those of their affiliated organizations, or those of the publisher, the editors and the reviewers. Any product that may be evaluated in this article, or claim that may be made by its manufacturer, is not guaranteed or endorsed by the publisher.
